# Excitatory and inhibitory effects of prolactin release activated by nerve stimulation in rat anterior pituitary

**DOI:** 10.1186/1477-7827-7-154

**Published:** 2009-12-31

**Authors:** Ping Zhang, Ling Liu, Cong-Jun Xie, Kai-Hu Wang, Li-Zhi Gao, Gong Ju

**Affiliations:** 1 Institute of Neurosciences, School of Life Sciences and Biotechnology, Shanghai Jiao Tong University, Shanghai, China; 2 Institute of Neurosciences, the Fourth Military Medical University, Xi'an, China; 3 School of Foreign Languages, Shanghai Jiao Tong University, Shanghai, China

## Abstract

**Background:**

A series of studies showed the presence of substantial amount of nerve fibers and their close relationship with the anterior pituitary gland cells. Our previous studies have suggested that aside from the classical theory of humoral regulation, the rat anterior pituitary has direct neural regulation on adrenocorticotropic hormone release. In rat anterior pituitary, typical synapses are found on every type of the hormone-secreting cells, many on lactotrophs. The present study was aimed at investigating the physiological significance of this synaptic relationship on prolactin release.

**Methods:**

The anterior pituitary of rat was sliced and stimulated with electrical field in a self-designed perfusion chamber. The perfusate was continuously collected in aliquots and measured by radioimmunoassay for prolactin levels. After statistic analysis, differences of prolactin concentrations within and between groups were outlined.

**Results:**

The results showed that stimulation at frequency of 2 Hz caused a quick enhancement of prolactin release, when stimulated at 10 Hz, prolactin release was found to be inhibited which came slower and lasted longer. The effect of nerve stimulation on prolactin release is diphasic and frequency dependent.

**Conclusions:**

The present in vitro study offers the first physiological evidence that stimulation of nerve fibers can affect prolactin release in rat anterior pituitary. Low frequency stimulation enhances prolactin release and high frequency mainly inhibits it.

## Background

It has been well acknowledged that the anterior pituitary is regulated via hypothalamic hormones released at the median eminence [1]. Although there are small amount of autonomic vascular nerve fibers in the gland, they do not play a role of direct regulation of hormone secretion [2,3]. Since the discovery of the presence of substantial amount of nerve fibers among the anterior pituitary gland cells of monkey in late 1980s [4,5], a series of studies have been conducted showing that most of these nerve fibers are closely related to the gland cells in several mammalian species [6-10], with their varicosities in close proximity to the latter. Electron microscopic studies have proven that the nerve terminals form synapses with the anterior pituitary gland cells, which serves as a golden morphological criterion that the nerve fibers can regulate the activity of gland cells [11,12]. In the rat, typical asymmetrical synapse can be found on every type of gland cells [13]. To evaluate the importance of synapses, their number per anterior pituitary of the rat has been quantitatively studied, demonstrating that there are as many as about 12,000 synaptophysin-like immunoreactive nerve terminals or varicosities in an anterior pituitary [14]. Further functional morphological studies by adrenalectomy [15-17] or ovariectomy [18] to manipulate the plasma hormone levels in the rat have shown profound increase in the density of nerve fibers in the anterior pituitary as a result of active axonal sprouting. The number of synapses was also found markedly increased after adrenalectomy [14]. Paden et al. reported that after adrenalectomy the sprouting nerve fibers tend to gather around the corticotrophs [19]. All these lines of evidence imply a direct neural regulation of the mammalian anterior pituitary and a hypothesis of neural-humoral dual regulation of the anterior pituitary has been postulated [11-13].

Having observed synapses on corticotrophs [11-13], we speculated that the nerve fibers might play the role of inducing rapid release of ACTH when a situation, such as acute stress, might call for. Our subsequent study indeed proved that when the nerve fibers in the anterior pituitary were stimulated at a low frequency of 2 Hz, which is known to induce release of classical transmitters from the small clear synaptic vesicles [20,21], there appeared an almost immediate surge of ACTH release of two minutes (min). However, when stimulated at 10 Hz, a frequency acknowledged to induce exocytosis of the large dense-core synaptic vesicles, which contain mainly neuropeptides [20,21]; there appeared a slower and longer gentle curve of ACTH inhibition [22,23].

In the rat, synapses are found on every hormone-secreting cell type. In fact, it is the lactotroph that is most frequently found to have a synapse [13]. Prolactin is another hormone hormone whose rapid release may be needed. It is clear that prolactin secretion is dramatically affected by stress. A myriad of stresses have been used to characterize such effects on prolactin secretion. These include but not limited to ether stress [24], restraint stress [25], thermal stress [26], hemorrhage [27], social conflict [28], and even academic stress in humans [29]. We speculated that nerve fibers might play a role of inducing rapid prolactin release. The present experiment was thus aimed to study the effect of nerve stimulation on prolactin release.

## Methods

### Animals

Adult male Sprague-Dawley rats, weighing 220-240 g, were obtained from FMMU's University Laboratory Animal Center. They were housed (three rats per cage) in a temperature-controlled room, and a 12: 12 h light/dark cycle (lights on at 07.00 h), with free access to food and water.

The rats were handled by holding and gripping 10 times each day for 5 consecutive days before experiments to minimize the effect of stress. Every precaution was taken to ensure proper and humane treatment of the rats. The number of rats used in each experiment group is indicated in the figure legends and tables.

### Tissue preparation

The experiments started at 09.00 h, the rats were anaesthetized with sodium pentobarbital (40 mg/kg body weight, i. p). After decapitation, the pituitary gland was immediately dissected. The intermediate and posterior lobes were then removed and discarded. The completeness of the removal was checked by hematoxylin and eosin (H-E) staining. The isolated anterior lobe was kept in pre-cooled (4°C) Krebs-Ringer bicarbonate solution (KRBGA, mmol/L: NaCl 119.0, KCl 4.7, CaCl_2 _2.5, KH_2_PO_4 _1.2, MgSO_4 _1.2, NaHCO_3 _25.0, Glucose 11.0, plus BSA 0.1% and bacitracin 40 mg/L) and rinsed three times. The anterior pituitary was then cut sagittally into 0.8 mm slices with a self-designed tissue chopper. The slices were subsequently transferred into a self-designed stimulation-perfusion chamber.

### Perfusion and electrical field stimulation (EFS)

The schematic diagram of the perfusion chamber was showed in our previous paper [23]. Within the perfusion chamber, the anterior pituitary slices were placed onto wide-bore nylon net between two flat ring-shaped platinum wire electrodes of 4 mm in diameter. The horizontally placed paired electrodes were 7 mm apart and had no contact with the nylon net and pituitary slices. The perfusion chamber was kept at 37°C with a surrounding water bath. The tissue slices were perfused at a rate of 0.1 ml/min with KRBGA (37°C), which was saturated with 95% oxygen and 5% carbon dioxide. After stabilization for 30 minutes (min), the perfusate was continuously sampled as aliquots (every 10 min or every 1 min) and stored at -20°C for RIA later. For 10-min sampling, the chamber volume was 0.9 ml and the dead volume of the perfusion system was 86 μl. For 1-min sampling, the chamber was made smaller in size with a volume of 0.15 ml, so that changes in prolactin secretion may be more quickly reflected in the perfusate.

The EFS was produced using a SEN 3001 stimulator (Nihon Kohden, Japan) and applied through two ring-shaped electrodes sandwiching the tissue slices. Based on our previous experience [22], square waves of 0.5 ms and 20 mA were used. 2 Hz and 10 Hz were selected for low and high frequency, respectively, which may induce different transmitter releasing [20,21]. The isolator function was chosen to produce trains of electrical stimulation and the whole stimulation output was continuously monitored through an oscilloscope.

### Prolactin RIA and statistic analysis

The double-antibody rat prolactin RIA kit (the rat prolactin antigen and antiserum were kindly provided by Dr. A. F. Parlow of National Institute of Diabetes and Digestive and Kidney Diseases, NIDDK, Torrance, CA, USA; and the radio-iodination was performed by Medical and Pharmacal Union Inc., Tianjin, China) was used for the measurement of prolactin concentrations. The range of assay was 10-200 pg/ml, with the intra- and inter-assay variation being 5.6% and 8.9% respectively. Measurements in duplicates were performed.

For statistic analysis, SPSS statistical software, version 10.0 (SPSS Inc., Chicago, IL, USA) was used. Significance of differences was determined by analysis of variance (ANOVA). Differences in changes of prolactin concentration within groups were determined by the *Dunnett t *-test. Differences between groups were determined by the least significant difference test (LSD). *P *< 0.05 was considered statistically significant (* *P *< 0.05, ** *P *< 0.01).

## Controls

### Perfusion controls

To test the basal release of prolactin during the experiment, the anterior pituitary slices were perfused with KRBGA only and the perfusate was continuously collected every 10 min or every 1 min.

### Tetrodotoxin (TTX) perfusion controls

To test the effect of TTX alone on prolactin secretion, TTX (purity 99.5%, supplied by the Shanghai Institute of Physiology) of 1 μmol/L was added to KRBGA after stabilization with KRBGA perfusing for 30 min. The perfusate was continuously collected every 10 min or every 1 min.

### TTX blocking controls

To block the action potential along the nerve fibers, TTX of 1 μmol/L was added to KRBGA, and the anterior pituitary slices were pre-incubated for 30 min before EFS started. To compare with the experimental groups, the perfusate was also continuously collected every 10 min or every 1 min.

### Functional viability controls

For assessment of the functional viability of lactotrophs during the experiment, KRBGA was replaced with high potassium KRBGA (KRBGA with 60.0 mmol/L of K^+ ^and 64.9 mmol/L of Na^+^) at the end of each test, to evoke peak prolactin output. To detect the concentration of prolactin, these samples were diluted with 0.85% NaCl solution before RIA.

## Results

### Perfusion controls

**Figure 1:**
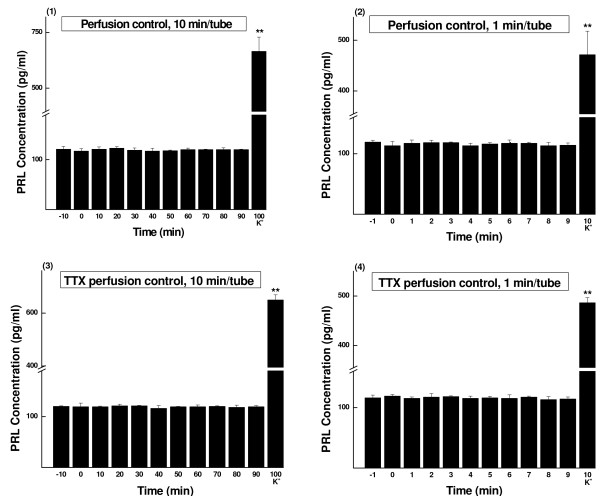
**PRL concentrations of sampled perfusate (Mean ± SD, pg/ml)**. The perfusate was continuously collected as aliquots and measured by RIA. (1) Slices were perfused with KRBGA and the perfusate was sampled every 10 min (n = 5). (2) Slices were perfused with KRBGA and the perfusate was sampled every 1 min (n = 5). (3) TTX (1 μmol/L) perfusion control of (1) (n = 5). (4) TTX (1 μmol/L) perfusion control of (2) (n = 5). K^+^, prolactin release was evoked by high potassium (60 mmol/L). ** *P *< 0.01.

**Figure 2:**
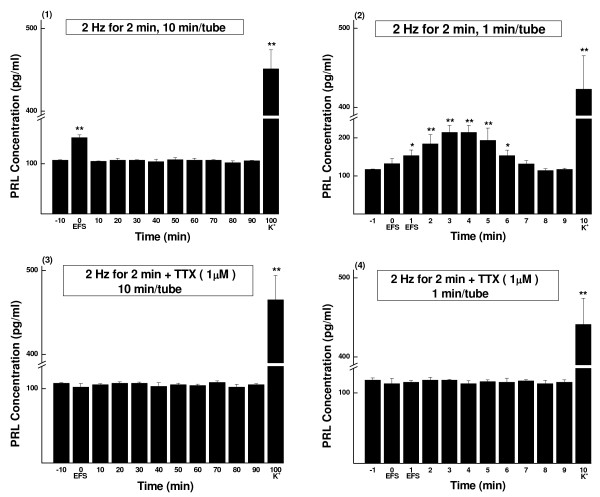
**PRL concentrations of sampled perfusate, EFS of 2 Hz for 2 min (Mean ± SD, pg/ml)**. The parameters of EFS were 20 mA, 0.5 ms and 2 Hz. The perfusate was continuously collected as aliquots and measured by RIA. (1) Perfusate sampled every 10 min (n = 4). (2) Perfusate sampled every 1 min (n = 6). (3) TTX (1 μmol/L) blocking control of (1) (n = 4). (4) TTX (1 μmol/L) blocking control of (2) (n = 5). K^+^, prolactin release was evoked by high potassium (60 mmol/L). * *P *< 0.05, ** *P *< 0.01.

After 30 min equilibration, the anterior pituitary slices were perfused with KRBGA only and the perfusate was continuously collected every 10 min (Fig. 1-1) or every 1 min (Fig. 1-2). No significant changes in prolactin basal levels were noticed during the experiment.

### TTX perfusion controls

**Figure 3:**
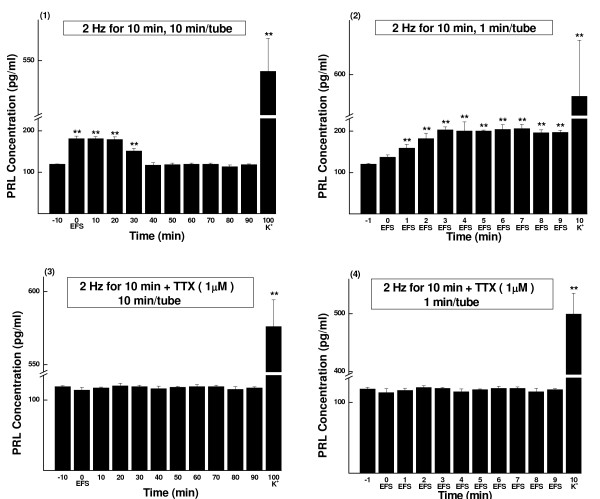
**PRL concentrations of sampled perfusate, EFS of 2 Hz for 10 min (Mean ± SD, pg/ml)**. The parameters of EFS were 20 mA, 0.5 ms and 2 Hz. The perfusate was continuously collected as aliquots and measured by RIA. (1) Perfusate sampled every 10 min (n = 6). (2) Perfusate sampled every 1 min (n = 5). (3) TTX (1 μmol/L) blocking control of (1) (n = 5). (4) TTX (1 μmol/L) blocking control of (2) (n = 6). K^+^, prolactin release was evoked by high potassium (60 mmol/L). ** *P *< 0.01.

**Figure 4:**
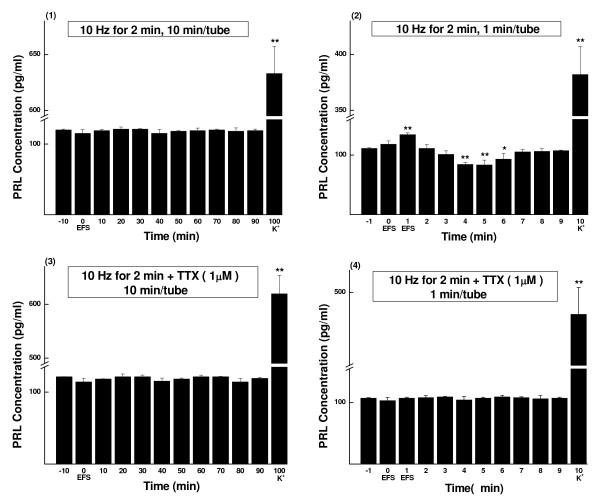
**PRL concentrations of sampled perfusate, EFS of 10 Hz for 2 min (Mean ± SD, pg/ml)**. The parameters of EFS were 20 mA, 0.5 ms and 10 Hz. The perfusate was continuously collected as aliquots and measured by RIA. (1) Perfusate sampled every 10 min (n = 5). (2) Perfusate sampled every 1 min (n = 5). (3) TTX (1 μmol/L) blocking control of (1) (n = 5). (4) TTX (1 μmol/L) blocking control of (2) (n = 4). K^+^, prolactin release was evoked by high potassium (60 mmol/L). * *P *< 0.05, ** *P *< 0.01.

After 30 min equilibration, the anterior pituitary slices were perfused with KRBGA containing TTX of 1 μmol/L and the perfusate was continuously collected every 10 min (Fig. 1-3) or every 1 min (Fig. 1-4). TTX of 1 μmol/L failed to affect prolactin basal secretion. No significant changes were noticed during the experiment.

### TTX blocking controls

**Figure 5:**
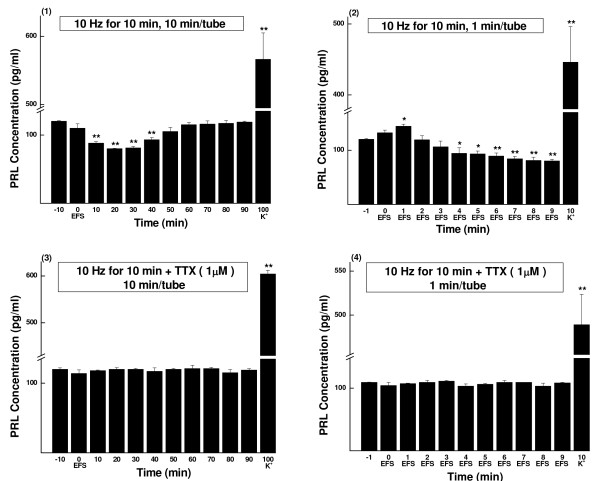
**PRL concentrations of sampled perfusate, EFS of 10 Hz for 10 min (Mean ± SD, pg/ml)**. The parameters of EFS were 20 mA, 0.5 ms and 10 Hz. The perfusate was continuously collected as aliquots and measured by RIA. (1) Perfusate sampled every 10 min (n = 5). (2) Perfusate sampled every 1 min (n = 7). (3) TTX (1 μmol/L) blocking control of (1) (n = 5). (4) TTX (1 μmol/L) blocking control of (2) (n = 4). K^+^, prolactin release was evoked by high potassium (60 mmol/L). * *P *< 0.05, ** *P *< 0.01.

TTX blocking controls showed that in every experiment either the excitatory or inhibitory effect of EFS could be totally blocked (see Figs. 2, 3, 4 and 5 and Tables 1, 2, 3 and 4).

**Table 1:** PRL concentrations of 1-min sampled perfusate, EFS of 2 min (Mean ± SD, pg/ml)

	Group A	Group B	Group C	Group D
**-1**	117 ± 1.0	118 ± 3.3	111 ± 1.3	107 ± 1.0
**0**	*132 ± 13.0 *	*113 ± 7.4 *	*118 ± 5.7 *	*103 ± 5.1 *
**1**	153 ± 15.1 *	*115 ± 2.6 *	134 ±3.3 **	*107 ± 1.7 *
**2**	184 ± 24.5**	118 ± 4.3	111 ± 6.6	108 ± 3.2
**3**	214 ± 18.6**	118 ± 1.2	101 ± 6.0	109 ± 1.0
**4**	214 ± 18.4**	113 ± 4.3	84 ± 3.9**	104 ± 5.8
**5**	193 ± 32.8**	116 ± 2.6	83 ± 8.6**	107 ± 1.5
**6**	153 ± 14.9*	115 ± 5.9	93 ± 9.6*	109±2.8
**7**	131 ± 8.4	117 ± 2.0	105 ± 4.6	108 ± 1.3
**8**	114 ± 5.3	113 ± 5.0	106 ± 4.6	106 ± 5.3
**9**	117 ± 3.2	115 ± 3.7	107 ± 1.3	107 ± 1.7
**K^+^**	423 ± 42.3**	441 ± 33.4**	382 ± 25.0**	470 ± 35.7**
**N**	6	5	5	4

Group A: 2 Hz. Group B: 2 Hz + TTX (1 μmol/L). Group C: 10 Hz. Group D: 10 Hz + TTX (1 μmol/L).

The perfusate was continuously collected as aliquots every 1 min and measured by RIA. Italic data represents results during electrical field stimulation.

TTX, tetrodotoxin; EFS, electrical field stimulation; K^+^, prolactin release was evoked by high potassium (60 mmol/L); N, the number of the rat used.

*Significant changes within group (* *P *< 0.05, ** *P *< 0.01).

**Table 2:** PRL concentrations of 1-min sampled perfusate, EFS of 10 min (Mean ± SD, pg/ml)

	Group E	Group F	Group G	Group H
**-1**	120 ± 2.1	119 ± 2.5	120 ± 1.6	109 ± 0.5
**0**	*137 ± 5.7 *	*114 ± 5.6 *	*132 ± 5.1 *	*104 ± 5.1 *
**1**	159 ± 9.3**	*117 ± 3.1 *	144 ± 4.1*	*107 ± 0.8 *
**2**	182 ± 13.0**	*121 ± 2.8 *	119 ± 7.6**	*109 ± 3.3 *
**3**	203 ± 7.4**	*120 ± 1.4 *	106 ± 11.0**	*111 ± 1.3 *
**4**	200 ± 22.5**	*115 ± 4.2 *	94 ± 10.7*	*103 ± 3.6 *
**5**	200 ± 2.6**	*118 ± 1.2 *	93 ± 5.3*	*106 ± 1.6 *
**6**	204 ± 11.7**	*120 ± 3.0 *	89 ± 6.0**	*109 ± 3.4 *
**7**	206 ± 9.9**	*120 ± 2.1 *	84 ± 4.6**	*109 ± 0.1 *
**8**	196 ± 7.4**	*115 ± 5.0 *	81 ± 6.1**	*103 ± 4.9 *
**9**	197 ± 5.3**	*118 ± 1.7 *	80 ± 3.3**	*108 ± 1.2 *
**K^+^**	568 ± 82.6**	499 ± 36.2**	446 ± 50.4**	489 ± 34.6**
N	5	6	7	4

Group E: 2 Hz. Group F: 2 Hz + TTX (1 μmol/L). Group G: 10 Hz. Group H: 10 Hz + TTX (1 μmol/L).

The perfusate was continuously collected as aliquots every 1 min and measured by RIA. Italic data represents results during electrical field stimulation.

TTX, tetrodotoxin; EFS, electrical field stimulation; N, the number of the rat used.

*Significant changes within group (* *P *< 0.05, ** *P *< 0.01).

**Table 3:** PRL concentrations of 10-min sampled perfusate, EFS of 2 min (Mean ± SD, pg/ml)

	Group I	Group J	Group K	Group L
**-10**	107 ± 1.0	107 ± 1.0	120 ± 1.1	121 ± 0.6
**0**	152 ± 5.9**	*102 ± 4.9 *	*115 ± 5.7 *	*114 ± 5.2 *
**10**	105 ± 0.5	105 ± 1.7	119 ± 1.7	118 ± 0.7
**20**	107 ± 3.9	107 ± 2.2	121 ± 3.2	121 ± 4.0
**30**	107 ± 1.0	107 ± 1.9	121 ± 1.1	121 ± 2.8
**40**	104 ± 5.2	103 ± 4.9	115 ± 5.9	115 ± 4.4
**50**	108 ± 4.0	105 ± 2.2	118 ± 1.3	118 ± 1.5
**60**	107 ± 3.7	108 ± 2.1	119 ± 3.7	121 ± 2.5
**70**	107 ± 1.3	108 ± 2.5	120 ± 1.1	121 ± 0.8
**80**	102 ± 3.9	102 ± 3.7	118 ± 5.0	114 ± 5.0
**90**	106 ± 0.8	105 ± 1.7	119 ± 2.4	119 ± 1.6
**K^+^**	451 ± 23.2**	465 ± 29.0**	633 ± 24.0**	619 ± 34.7**
**N**	4	4	5	5

Group I: 2 Hz. Group J: 2 Hz + TTX (1 μmol/L). Group K: 10 Hz. Group L: 10 Hz + TTX (1 μmol/L).

The perfusate was continuously collected as aliquots every 10 min and measured by RIA. Italic data represents results during electrical field stimulation.

TTX, tetrodotoxin; EFS, electrical field stimulation; N, the number of the rat used.

*Significant changes within group (** P < 0.01).

**Table 4:** PRL concentrations of 10-min sampled perfusate, EFS of 10 min (Mean ± SD, pg/ml)

	Group M	Group N	Group O	Group P
**-10**	119 ± 1.0	119 ± 1.3	120 ± 1.2	120 ± 2.1
**0**	181 ± 6.1**	*114 ± 3.8 *	*110 ± 6.6 *	*114 ± 5.2 *
**10**	181 ± 5.1**	117 ± 1.3	88 ± 2.4**	118 ± 1.1
**20**	179 ± 6.4**	120 ± 3.6	80 ± 0.6**	120 ± 2.4
**30**	151 ± 7.0**	119 ± 2.0	81 ± 2.3**	120 ± 1.3
**40**	117 ± 6.0	116 ± 3.7	93 ± 3.3**	117 ± 5.2
**50**	118 ± 4.2	118 ± 1.5	105 ± 6.3	120 ± 1.1
**60**	119 ± 3.2	119 ± 3.0	115 ± 3.6	121 ± 4.8
**70**	119 ± 3.2	119 ± 1.9	116 ± 4.7	121 ±1.7
**80**	113 ± 4.4	115 ± 3.9	117 ± 4.5	115 ± 4.6
**90**	118 ± 2.1	117 ± 1.6	119 ± 1.4	119 ± 2.1
**K^+^**	542 ± 24.4**	576 ± 18.3**	566 ± 38.9**	604 ± 8.3**
**N**	6	5	5	5

Group M: 2 Hz. Group N: 2 Hz + TTX (1 μmol/L). Group O: 10 Hz. Group P: 10 Hz + TTX (1 μmol/L).

The perfusate was continuously collected as aliquots every 10 min and measured by RIA. Italic data represents results during electrical field stimulation.

TTX, tetrodotoxin; EFS, electrical field stimulation; N, the number of the rat used.

*Significant changes within group (** P < 0.01).

### Functional viability controls

At the end of each test, high potassium caused drastic secretion of prolactin (see Figs. 1, 2, 3, 4 and 5). The results indicated good secretory responsiveness of lactotrophs and showed the viability of the gland cells during the entire experiment.

### Effects of low frequency (2 Hz) stimulation on prolactin release

#### Stimulation of 2 min

The EFS started at the beginning of sample-0. When analyzed in 10-min samples (Fig. 2-1 and Group I of Table 3), there was a brief sharp increase in prolactin secretion, detectable only at sample-0 (in average, 142% of the basal secretion level, *P *< 0.01). In 1-min samples (Fig. 2-2 and Group A of Table 1), a wave of prolactin secretion lasting for 6 min (from sample-1 to sample-6) was noticed, which started from the second min of stimulation (sample-1, 131%, *P *< 0.05), peaked at sample-3 and sample-4 (both 183%, *P *< 0.01), and then subsided until sample-7, when the prolactin secretion returned to the basal level.

#### Stimulation of 10 min

The 1-min samples showed that the rising phase of the increase appeared the same as that in the 2-min stimulation experiment. After reaching the peak at sample-3 (169%, P < 0.01), prolactin secretion level maintained as a plateau until the end of the 10-min experiment (Fig. 3-2 and Group E of Table 2). When assayed in 10-min samples (Fig. 3-1 and Group M of Table 4), the increase in prolactin secretion continued for about 30 min. At samples 0-20, the relative secretion levels were 152%, 152% and 150%, respectively (*P *< 0.01 for all). At sample-30, it declined to 127% (*P *< 0.01) and then returned to the basal level at sample-40 (Fig. 3-1 and Group M of Table 4). No inhibition of prolactin release was observed in the 2-Hz stimulation groups.

Statistic analysis proved that there was significant difference between 2-min and 10-min stimulation groups (*P *< 0.01).

### Effects of high frequency (10 Hz) stimulation on prolactin release

#### Stimulation of 2 min

When the anterior pituitary slices were stimulated for 2 min and examined in 10-min samples, no significant change in prolactin release could be detected (Fig. 4-1 and Group K of Table 3). But when parceled in 1-min samples (Fig. 4-2 and Group C of Table 1), it became evident that there was an immediate transient increase in prolactin release only at sample-1 (121%, *P *< 0.01), which was followed by an inhibition of 3 min, from sample-4 to sample-6 (76%, *P *< 0.01; 75%, *P *< 0.01; and 84%, *P *< 0.05; respectively). The prolactin concentration resumed basal level from sample-7 on (Fig. 4-2 and Group C of Table 1).

#### Stimulation of 10 min

In 1-min samples (Fig. 5-2 and Group G of Table 2), an early 1-min only transient increase of prolactin release appeared (sample-1, 120%, *P *< 0.05), the same as in 2-min stimulation experiment. Similarly, the inhibition started from sample-4, but it lasted till the end of the experiment (sample-9) with a very gentle down slope (sample-4 to sample-9: 78%, *P *< 0.05; 78%, *P *< 0.05; 74%, *P *< 0.01; 70%, *P *< 0.01; 68%, *P *< 0.01; 67%, *P *< 0.01; respectively). The 10-min sample analysis (Fig. 5-1 and Group O of Table 4) revealed that the suppression continued for 40 min, from sample-10 to sample-40 (73%, 67%, 68%, 78%, respectively; *P *< 0.01 for all), with the lowest value at samples-20 and 30.

Statistic analysis showed that there was significant difference between 2-min and 10-min stimulation groups (*P *< 0.01).

## Discussion

Several groups reported that the anterior pituitary contains calcitonin gene-related peptide [30-32] and substance P [33] immunoreactive nerve fibers. Reports of Gao LZ et al. have strongly implied the existence of direct neural regulation on ACTH release [22,23]. The present study indicates that EFS of the nerve fibers in the anterior pituitary of the rat have excitatory and inhibitory effects on prolactin secretion.

In the present experiment, it is most important to confirm that nerve fibers are stimulated directory and the gland cells are not done. The reversing effect of TTX on the electrically evoked changes suggests an indirect effect of the stimulus on the lactotrophs. TTX itself has no effect on lactotrophs of prolactin release, and at the same time, the existence of TTX could totally block the changes of lactotrophs releasing caused by EFS. It is safe to conclude that the lactotrophs are not stimulated directly. The only reasonable explaination is that nerve fibers are stimulated directly. The fluctuation of the prolactin level is the result of nerve releasing.

The anterior pituitary is composed of five major hormone-secreting cell types, corticotrophs, lactotrophs, thyrotrophs, somatotrophs and gonadotrophs. These cells are of the same origin, but exhibit marked differences in the expression levels of the ionic channels and differ with respect to their patterns of basal hormone secretion [34]. Specifically, lactotrophs exhibited low expression levels of TTX-sensitive Na^+ ^channel, which may be of physiological significance for the control of Ca^2+ ^homeostasis and secretion of prolactin. Sankaranarayanan S et al. reported that TTX had no effect on spontaneous action potentials of primary cultured lactotrophs [35]. Kazahari K et al. reported that voltage-sensitive sodium channels are not expressed in the anterior pituitary of rat [36]. In our present experiment, TTX (1 μmol/L) itself did not affect the secretion level of lactotrophs and no significant change in prolactin release was detected when perfused with KRBGA containing 1μmol/L TTX only. In every experiment, both the excitatory and inhibitory effect of EFS could be totally blocked by TTX suggesting that direct stimulation of the gland cells was unlikely, which is also supported by the biphasic nature of prolactin secretion following 10 Hz stimulation.

Neurotransmitters pass messages from one particular cell to another, which happens in a particular time and a particular place [37]. In the present experimental system, during and after EFS of 2 Hz, the releasing of neurotransmitter(s) is the dominant reason for the fluctuation of prolactin level. Peptides are public announcements, messages endure, at least for a while; they are messages from one population of neurons to another [38]. During and after EFS of 10 Hz, neurotransmitters (in smaller quantity) and peptides (in greater quantity) worked together, volume transmission or a paracrine effect may well play a role at the same time. These combined effects may cause the fluctuation of the perfusate prolactin level.

The neuroendocrine control of prolactin secretion is different from that of any other pituitary hormone [39]. It is predominantly inhibited by the hypothalamus [40] and acts directly in the brain to suppress its own secretion via a system known as short-loop feedback [41,42]. Is direct neural regulation another way of inhibition? Lactotrophs have a high basal activity and spontaneously secret prolactin into the blood. In this in vitro study the lactotrophs were freed from the tonic hormonal inhibition in vivo, thus assuming a status of basal secretion. It is interesting, therefore, to see what may happen if the nerve stimulation is applied when the lactotrophs are under hormonal regulation. Actually, the effect of stimulation of nerve fibers on the anterior pituitary in vivo is an issue very difficult to study, because it is almost impossible to rule out the concomitant stimulation on gland cells, or the induction of hypothalamic hormonal changes.

Both prolactin and ACTH may be released in great amount in urgent situations, but differ remarkably in their patterns in the experiment. It is therefore not surprising to find that EFS of 2 Hz, which causes the release of classical transmitters [20,21], can induce immediate secretion changes in both prolactin and ACTH. Although the release of prolactin occurred as quickly as in the case of ACTH, it followed a much gentler slope and lasted longer. When stimulated for 10 min, the enhancing effect on prolactin release lasted for as long as 30 min, whereas for ACTH, there was only sharp peak of release even though the stimulation continued for 10 min [22]. The reason of this difference remains to be elucidated. It may indicate that there are different functional needs for immediate release of prolactin. Apparently, the neuronal firing pattern may well be different for different needs, resulting in different secretion pattern.

EFS 10-Hz also gave a very different response curve from that of the ACTH. The interesting point is that, when stimulated for 10 min at 10 Hz, the prolactin response started with a brief increase in prolactin release of 1 min, and then inhibition began 3 min later and lasted for about 40 min. In the case of ACTH, 10-Hz stimulation caused an inhibition of ACTH release only. Arguably, this is caused by its serving as a quick and precise negative feedback for the humoral enhancement of its release. Ten-Hz stimulation is known to cause release of neuropeptide(s) along with a smaller quantity of classical transmitter [20,21]. We believe the initial brief increase is due to the co-release of classical transmitter(s). Due to its small quantity, the response was much weaker than that in the 2-Hz stimulation experiment.

## Conclusions

The present in vitro study offers the first physiological evidence that stimulation of nerve fibers can affect prolactin release in rat anterior pituitary. Low frequency stimulation enhances prolactin release and high frequency mainly inhibits it.

## Abbreviations

KRBGA: Krebs-Ringer bicarbonate solution; EFS: electrical field stimulation; TTX: tetrodotoxin; RIA: radioimmunoassay; min: minute(s); PRL: prolactin; ACTH: adrenocorticotropic hormone.

## Competing interests

The authors declare that they have no competing interests.

## Authors' contributions

PZ carried out the main experiments and wrote the first draft of the manuscript. LL performed the RIA. CJX performed the statistical analysis. KHW structured and proofread the final manuscript. LZG and GJ designed the study and wrote the final manuscript. All authors read and approved the final manuscript.
